# Hospitalizations due to primary care sensitive conditions: an ecological study

**DOI:** 10.11606/S1518-8787.2019053000403

**Published:** 2018-12-20

**Authors:** Ludmila Grego Maia, Luiz Almeida da Silva, Rafael Alves Guimarães, Bruno Bordin Pelazza, Ana Cláudia Souza Pereira, Wender Lopes Rezende, Maria Alves Barbosa

**Affiliations:** I Universidade Federal de Goiás. Regional Jataí. Jataí, GO, Brasil; II Universidade Federal de Goiás. Regional Catalão. Catalão, GO, Brasil; III Universidade Federal de Goiás. Instituto de Patologia Tropical e Saúde Pública. Goiânia, GO, Brasil; IV Secretaria Municipal de Saúde de Jataí. Jataí, GO, Brasil; V Universidade Federal de Goiás. Faculdade de Enfermagem. Goiânia, GO, Brasil

**Keywords:** Primary Health Care, Basic Health Services, Health Care Quality, Access, and Evaluation, Triage, organization & administration, Atenção Primária à Saúde, Serviços Básicos de Saúde, Qualidade, Acesso e Avaliação da Assistência à Saúde, Triagem, organização & administração

## Abstract

**OBJECTIVE:**

To evaluate the trend of hospitalizations due to primary care sensitive conditions and its relationship with the Family Health Strategy coverage.

**METHODS:**

Ecological study of time series using the records from the Hospital Information System, from 2005 to 2015, with data for the state of Goiás, Brazil. Trend analyses were performed by the generalized linear regression method of Prais-Winsten with robust variance, which allowed to verify if the trend of hospitalizations due to primary care sensitive conditions was stationary (p > 0.05), declining (p < 0.05 and negative regression coefficient), or ascending (p < 0.05 and positive regression coefficient) in each region of Goiás and for each diagnosis group, stratified by sex. Pearson correlation was used to verify the degree of association between the Family Health Strategy coverage and the rate of hospitalizations due to primary care sensitive conditions.

**RESULTS:**

Hospitalizations due to primary care sensitive conditions accounted for 1,092,070 (30.0%) of hospitalizations in Goiás. The average hospitalizations rate due to primary care sensitive conditions was statically less than the rate for other conditions in the analyzed period (167.6% against 386.2%; t = −13.18; p < 0.001). There has been a downward trend in hospitalizations trend due to primary care sensitive conditions in Goiás and in most health regions. The trends varied between sexes in the groups of causes. We observed a negative correlation between the Family Health Strategy coverage and the hospitalizations trend due to primary care sensitive conditions in the state and also in most health regions.

**CONCLUSIONS:**

Hospitalizations due to primary care sensitive conditions had a significant reduction trend over the analyzed period. Despite this progressive decrease, this rate remains high and the reduction trend was not linear for all causes. These results allow for directing public policies, while drawing a general overview of hospitalizations due to primary care sensitive conditions by sex and region in the state.

## RESUMO

**OBJETIVO:**

Avaliar a tendência das internações por condições sensíveis à atenção primária e sua relação com a cobertura da Estratégia Saúde da Família.

**MÉTODOS:**

Estudo ecológico de séries temporais utilizando registros do Sistema de Informação Hospitalar referentes ao período de 2005 a 2015, com dados para o estado de Goiás. As análises de tendência foram realizadas por regressão linear generalizada pelo método de Prais-Winsten com variância robusta, que permitiu verificar se a tendência de internações por condições sensíveis à atenção primária foi estacionária (p > 0,05), declinante (p < 0,05 e coeficiente de regressão negativo) ou ascendente (p < 0,05 e coeficiente de regressão positivo) em cada região de Goiás e por grupo diagnóstico estratificado por sexo. A correlação de Pearson foi utilizada para verificar o grau de associação entre cobertura da Estratégia Saúde da Família e a taxa de internações por condições sensíveis à atenção primária.

**RESULTADOS:**

As internações por condições sensíveis à atenção primária foram responsáveis por 1.092.070 (30,0%) internações em Goiás. A taxa média de internações por condições sensíveis à atenção primária foi estaticamente menor que a taxa por outras condições no período analisado (167,6% *versus* 386,2%; *t* = −13,18; p < 0,001). Verificou-se uma tendência decrescente das taxas de internações por condições sensíveis à atenção primária em Goiás e na maioria das regiões de saúde. As tendências variaram entre os sexos nos grupos de causas. Observou-se correlação negativa entre a cobertura da Estratégia Saúde da Família e a taxa de internações por condições sensíveis à atenção primária no estado e também na maioria das regiões de saúde.

**CONCLUSÕES:**

As internações por condições sensíveis à atenção primária tiveram tendência de redução significativa ao longo do período analisado. Apesar dessa diminuição progressiva, essa taxa permanece elevada e a tendência de redução não foi linear para todas as causas. Estes resultados possibilitam direcionar políticas públicas, ao desenhar um panorama geral das internações por condições sensíveis à atenção primária por sexo e região no estado.

## INTRODUCTION

Since the implementation of the Brazilian Unified Health System (SUS), health actions have been carried out to consolidate its fundamental principles and guidelines. Mostly from the 1990s, the Ministry of Health (MS) has sought to extend and strengthen primary health care (PHC) through technical studies, aiming to develop health promotion, prevention, and recovery actions within the collective context. This initiative is based on successful experiences in various countries and on principles from the World Health Organization (WHO). It is believed that the actions of PHC have great ability to modify the population's profile of morbidity and mortality, consequently improving the quality of life of citizens and health indicators^1^ ^–^ ^3^.

In Brazil, PHC is the first level of care, with the main purpose of organizing forms to access the health system^3^. This conception was reaffirmed by Decree 7,508, of 2011, that established it as organizer of the universal and equal access as well as a gateway to the other levels of SUS care^4^. The Family Health Strategy (FHS) was adopted to guide and standardize the actions of PHC, defining reference teams, territories of operation, and attributes of the work process^1^.

Systems organized from the PHC, especially those with good coverage (above 70%) by the FHS model, present lower hospitalization trends for some causes and considerable decrease of health costs^5^ ^–^ ^7^.

Several indicators were developed to assess the efficaciousness of PHC. In the 1980s, in the United States, a study conducted by Billings and Teicholz^8^, aiming to identify the impact of lack of access to outpatient care, established the Ambulatory Care Sensitive Conditions indicator, which measures the potentially preventable hospitalizations and relates them to problems of effectiveness regarding primary care^8^. Since then, other studies have been developed with data from potentially preventable hospitalizations. In 2008, by Ordinance GM/MS 221, of April 17, the MS published the Brazilian list of primary care sensitive conditions (PCSC) and defined it as an instrument to evaluate PHC^9^ ^,^ ^10^.

We understand as sensitive conditions health injuries whose profile of morbidity and mortality can be reduced or modified by a resolutive and efficient primary care^8^ ^,^ ^9^. From this list, it is possible to estimate hospitalization rates due to primary care sensitive conditions (HPCSC) and evaluate the performance of health services, in addition to verifying the effectiveness of public policies. HPCSC rates are also used to evaluate the efficaciousness, quality, and accessibility of PHC^6^ ^,^ ^11^.

With the publication of the previously mentioned Ordinance, it is possible to observe an increase in Brazilian publications related to HPCSC^6^, something that was already happening in other countries^12^ ^,^ ^13^.

One of the major contributions of this indicator is to allow for health services to measure their ability to solve problems which could be cared for by PHC. For the diagnoses listed, there are timely and preventive measures, as well as outpatient treatments; in other words, they are preventable causes if cared for with precise, determined, and efficient actions by primary care teams^13^.

The HPCSC provide to the managing team a strategic and comprehensive view on the quality of primary care, enabling the visualization of gaps in the system. Many of them are known by a large part of health managers, such as: a) difficulty of associating health needs to the community's demands; b) lack of coordination between the levels of assistance; c) insufficiency and mismanagement of financial resources; d) precariousness of employment relations, generating large turnover of health professionals, especially physicians^2^ ^,^ ^3^.

Thus, considering the importance of PHC for SUS, this study aimed to evaluate the HPCSC trend in the state of Goiás, Brazil, in a historical series from 2005 to 2015, as well as its relationship with Family Health Strategy coverage.

## METHODS

This is an ecological study of time series concerned with the hospitalizations of residents of the state of Goiás, Brazil, due to primary care sensitive conditions, registered in the *Sistema de Internação Hospitalar* (SIH - Hospitalization System) from 2005 to 2015. Time series can be defined as organization models of certain quantitative information in time^14^.

Goiás is the 12th most populous state and the ninth largest economy among the Brazilian states. It is situated in the Midwest region, with a territorial extension of 340,086 km^2^, population of 6,695,855 inhabitants, human development index of 0.735 and per capita income of R$1,077.00^15^. The State has 246 municipalities, grouped into five macro health regions, subdivided in 18 regions: (i) Central; (ii) Center-South; (iii) North Surroundings; (iv) South Surroundings; (v) Estrada de Ferro; (vi) Northeast I; (vii) Northeast II; (viii) North; (ix) West I; (x) West II; (xi) Pirineus; (xii) Rio Vermelho; (xiii) Serra; (xiv) Southwest I; (xv) Southwest II; (xvi) South; (xvii) São Patrício I; and (xviii) São Patrício II^15^ ^,^ ^16^.

As for the FHS coverage in the state, data from the Department of Basic Care of the MS show that in the period of study there was a growth of 51.5% (2005) to 67.2% (2015), a total of 1,036 teams deployed in the cities of Goiás^17^.

To calculate HPCSC rates, the data were tabulated in the TabWin program version 4.1.1, and were selected having as a reference the *Caderno de Diretrizes, Objetivos, Metas e Indicadores* [Guidelines, Objectives, Goals, and Indicators Notebook] published by the MS^9^. National tables and definition files were downloaded from the website www.datasus.gov.br and stored to extract the data used in the research.

The causes of hospitalization and diagnosis analyzed are described in Ordinance GM/MS 221, of April 17, 2008, and present at the 10th International Classification of Diseases and Related Health Problems (ICD-10). They are: immunopreventable diseases and sensitive conditions (A33-A37, A95, B16, B05-B06, B26, G00.0, A17.0, A19, A15-A16, A18, A17.1-A17.9, I00-I02, A51-A53, B50-B54, and B77); infectious gastroenteritis and complications (E86 and A00-A09); anemia (D50); nutritional deficiencies (E40-E46 and E50-E64); ear, nose, and throat infections (H66, J00-J03, J06, and J31); bacterial pneumonias (J13-J14, J 15.3-J 15.4, J 15.8-J 15.9, and J 18.1); asthma (J45-J46); lower airway diseases (J20, J21, J40-J44, and J47); hypertension (110-111); angina (I20); heart failure (I50 and J81); cerebrovascular diseases (I63-I67, I69, and G45-G46); diabetes mellitus (E10-E14); epilepsy (G40-G41); kidney and urinary tract infections (N10-N12, N30, N34, and N39.0); skin and subcutaneous tissue infections (A46, L01-L04, and L08); female pelvic organs inflammatory disease (N70-N73 and N75-N76); gastrointestinal ulcer (K25-K28, K92.0, K92.1, and K92.2); prenatal and birth-related diseases (O23, A50, and P35.0); congenital syphilis (A50), and congenital rubella syndrome (P35.0), reported in both sexes^9^.

The data selected for tabulation in TabWin 4.1.1 program were: total population of the state of Goiás, obtained from the website of the Brazilian Institute of Geography and Statistics (IBGE), total number of hospitalizations (excluding child-birth), and total number of hospitalizations due to PCSC^15^. The SIH qualifies the health information from the record of attendances to users hospitalized in health establishments of SUS and provides reports and data from these hospitalizations^18^.

The results were analyzed in the statistical program Stata, version 12.0. Initially, HPCSC rates and of hospitalizations due to other sensitive conditions were calculated per year. The HPCSC rates in Goiás were calculated by the ratio between the total number of HPCSC and resident population in the year, multiplied by 10,000. The rates of hospitalizations due to other conditions were calculated by the ratio between the total number of hospitalizations, excluding child-birth, and the resident population in Goiás in each year, multiplied by 10,000. Both rates were standardized by the direct method. The average rates of HPCSC and hospitalizations due to other conditions during the analyzed period were compared using the Student's t-test for independent samples.

Then, the global HPCSC trends for the state and each health region described were analyzed. Analyses of HPCSC trends by diagnosis group and for the male and female sexes were also carried out. Global HPCSC rates and for each group were calculated by the ratio between the number of HPCSC per group and the resident population of each sex in a year, multiplied by 10,000. All rates were standardized by age and by the direct method and logarithmized.

Trend analyses of the historical series were performed by generalized linear regression by the Prais-Winsten method with robust variance, which allowed to verify if HPCSC trends were stationary (p > 0.05), declining (p < 0.05 and negative regression coefficient), or ascending (p < 0.05 and positive regression coefficient) in each region of Goiás and for each diagnosis group, stratified by sex - a method previously used by Boing et al.^18^ From the regression coefficient and standard error, the annual mean percentage variations of HPCSC rates were calculated.

In a second analysis, Pearson correlation was applied between HPCSC rates and FHS coverage^19^. Values of p < 0.05 were considered statistically significant.

## RESULTS

Between 2005 and 2015, 4,072,744 hospitalizations occurred in Goiás. Excluding the hospitalizations for child-birth (n = 407,908), the HPCSC accounted for 1,092,070 (30.0%) of the total number of hospitalizations in SUS. The Figure shows the rate of HPCSC per 10,000 inhabitants, rate of hospitalizations due to other conditions in Goiás and FHS population coverage between 2005 and 2015. The average rate of HPCSC was statically less than the rate oh hospitalizations due to other conditions in the analyzed period (167.58% against 386.25%; t = −13.18; p < 0.001).

**Figure:**
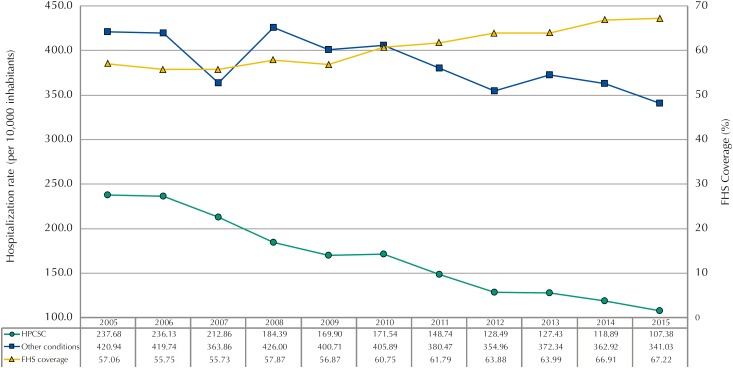
Hospitalization rate due to primary care sensitive conditions (HPCSC), hospitalization rate due to other conditions, and Family Health Strategy (FHS) coverage. Goiás, 2005-2015.

Table 1 presents the trend of HPCSC cases for the male sex, which was descending, with average annual variation of −17.1%. We also observed a reduction trend in most groups of causes. Among the causes, stood out the stability of immunopreventable diseases (β = 6.24; p = 0.302) and increase of ear, nose, and throat infections (β = 0.137; p = 0.013); angina (β = 0.030; p< 0.001); skin and tissue infections (β = 0.019; p = 0.043); and hospitalizations due to pre-natal and birth (β = 0.231; p = 0.004).

**Table 1:** Trend of hospitalizations rate for primary care sensitive conditions (HPCSC), according to the group of causes in the male sex. Goiás, 2005-2015.

HPCSC		Male sex				
		β^a^ (95%CI^b^)	R2	p	Average annual variance (95%CI^b^)	Interpretation
General		-0.081 (-0.089− -0.073)	0.974	< 0.001	– 1 7.12 (-18.57− -15.65)	Reduction
Immunopreventable diseases		0.026 (-0.028-0.080)	0.141	0.302	6.24 (-5.95-20.01)	Stability
Gastroenteritis		-0.115 (-0.135− -0.095)	0.966	< 0.001	-23.37 (-26.67− -19.92)	Reduction
Anemia		-0.116 (-0.136− -0.096)	0.787	< 0.001	-23.52 (-26.92− -19.96)	Reduction
Nutritional deficiencies		-0.057 (-0.074− -0.040)	0.908	< 0.001	-12.41 (-15.62− -9.07)	Reduction
Ear, nose, and throat infections		0.137 (0.037-0.237)	0.384	0.013	37.25 (9.62-71.83)	Increase
Bacterial pneumonia		-0.064 (-0.124− -0.005)	0.797	0.037	-13.88 (-24.69− -1.52)	Reduction
Asthma		-0.158 (-0.171− -0.145)	0.989	< 0.001	-30.63 (-32.65− -28.55)	Reduction
Lung diseases		-0.093 (-0.098− -0.088)	0.992	< 0.001	-1 9.38 (-20.33− -18.41)	Reduction
Hypertension		-0.178 (-0.219− -0.137)	0.962	< 0.001	-33.64 (-39.46− -27.25)	Reduction
Angina		0.030 (0.015-0.045)	0.644	< 0.001	7.34 (3.78-1 1.01)	Increase
Heart failure		-0.108 (-0.130− -0.086)	0.970	< 0.001	-22.06 (-25.79− -18.15)	Reduction
Cerebrovascular diseases		-0.039 (-0.052-0.077)	0.943	0.014	-6.68 (-1 1.22− -1.91)	Reduction
Diabetes mellitus		-0.054 (-0.084− -0.024)	0.947	0.003	-11.75 (-17.50− -5.58)	Reduction
Epilepsies		-0.049 (-0.064− -0.035)	0.876	< 0.001	-10.84 (-13.73− -7.84)	Reduction
Kidney and urinary tract infections		-0.022 (-0.040-0.004)	0.910	0.018	-5.1 0 (-8.83− -1.22)	Reduction
Skin and subcutaneous tissue infection		0.019 (0.000-0.038)	0.237	0.043	4.62 (0.30-9.12)	Increase
Female pelvic organs inflammatory disease						
	Gastrointestinal ulcer	-0.162 (-0.209-0.1 15)	0.921	< 0.001	-31.26 (-38.1 1− -23.1 1)	Reduction
	Prenatal and birth-related	0.231 (0.095-0.368)	0.656	0.004	70.56 (25.67-131.48)	Increase

a Regression coefficient.

b 95% confidence interval.

For the female sex, Table 2 shows that the HPCSC also presented a downward trend, with annual mean variation of −17.6%. Among the causes, stood out the stability of immunopreventable diseases (β = 0.036; p = 0.350) and ear, nose, and throat infections (β = 0.148; p = 0.013); and pre-natal and birth related-diseases (β = 0.094; p = 0.001).

**Table 2:** Trend of hospitalizations rate for primary care sensitive conditions (HPCSC), according to the group of causes in the female sex. Goiás, 2005-2015.

HPCSC	Female sex				
	β^a^ (95%CI^b^)	R2	p	Average annual variance (95%CI^b^)	Interpretation
General	-0.083 (-0.089-0.078)	0.986	< 0.001	-17.56 (-24.28− -10.25)	Reduction
Immunopreventable diseases	0.036 (-0.049-0.123)	0.077	0.359	8.89 (-1 0.31-32.72)	Stability
Gastroenteritis	-0.109 (-0.127− -0.090)	0.957	< 0.001	-22.21 (-25.34− -1 8.95)	Reduction
Anemia	-0.139 (-0.180− -0.098)	0.838	< 0.001	-27.51 (-33.87− -20.53)	Reduction
Nutritional deficiencies	-0.061 (-0.083− -0.039)	0.818	< 0.001	-13.26 (-1 7.55− -8.74)	Reduction
Ear, nose, and throat infections	0.148 (0.040-0.256)	0.407	0.013	40.74 (10.49-79.28)	Increase
Bacterial pneumonia	-0.069 (-0.129− -0.010)	0.755	0.026	-14.88 (-25.52− -2.72)	Reduction
Asthma	-0.160 (-0.169− -0.150)	0.993	< 0.001	-30.83 (-32.34− -29.28)	Reduction
Lung diseases	-0.094 (-0.107− -0.081)	0.963	< 0.001	-19.59 (-21.90− -1 7.22)	Reduction
Hypertension	-0.189 (-0.229− -0.149)	0.969	< 0.001	-35.37 (-40.85− -29.38)	Reduction
Angina	-0.028 (-0.047-0.008)	0.844	0.010	-6.26 (-10.27− -2.07)	Reduction
Heart failure	-0.116 (-0.131− -0.100)	0.977	< 0.001	-23.46 (-26.46− -20.72)	Reduction
Cerebrovascular diseases	-0.044 (-0.067-0.021)	0.949	0.002	-9.70 (-14.24− -4.92)	Reduction
Diabetes mellitus	-0.091 (-0.119− -0.064)	0.968	< 0.001	-19.07 (-23.86− -13.99)	Reduction
Epilepsies	-0.040 (-0.051− -0.029)	0.816	< 0.001	-8.91 (-9.92− -7.89)	Reduction
Kidney and urinary tract infections	-0.018 (-0.035− -0.002)	0.976	0.027	-4.26 (-7.65− -0.73)	Reduction
Skin and subcutaneous tissue infection	-0.006 (-0.025-0.012)	0.031	0.462	-1.50 (-5.68-2.86)	Stability
Female pelvic organs inflammatory disease	-0.099 (-0.130− -0.067)	0.909	< 0.001	-20.38 (-25.77− -14.61)	Reduction
Gastrointestinal ulcer	-0.162 (-0.190− -0.134)	0.962	< 0.001	-31.1 8 (-35.35− -26.74)	Reduction
Prenatal and birth-related	0.094 (0.048-0.140)	0.672	0.001	24.30 (12.19-3 7.72)	Increase

a Regression coefficient.

b 95% confidence interval.

HPCSC rate trends were also evaluated according to health region, as can be seen in Table 3. We observed a downward trend in Goiás, with annual mean variation of −17.4%, as well as in most health regions, except Northeast II and São Patrício II.

**Table 3:** Trend of hospitalizations rate for primary care sensitive conditions (HPCSC), according to the health region in Goiás, 2005-2015.

Region	HPCSC rate				
	p^a^ (95%CI^b^)	R2	p	Average annual variance (95%CI^b^)	Interpretation
Goiás	-0.083 (-0.089− -0.076)	0.982	< 0.001	-17.41 (-24.14− -10.08)	Reduction
Central	-0.088 (-0.106− -0.071)	0.992	< 0.001	-18.44 (-21.54− -15.21)	Reduction
Center-South	-0.075 (-0.093− -0.055)	0.964	< 0.001	-15.83 (-19.33− -12.1 7)	Reduction
North Surroundings	-0.080 (-0.109− -0.050)	0.837	< 0.001	-16.85 (-22.11− -1 1.24)	Reduction
South Surroundings	-0.179 (-0.259− -0.098)	0.783	0.001	-33.78 (-44.68− -20.75)	Reduction
Estrada de Ferro	-0.063 (-0.108− -0.019)	0.887	0.010	-13.67 (-21.83− -4.66)	Reduction
Northeast I	-0.147 (-0.220− -0.075)	0.930	0.001	-28.86 (-39.55− -1 6.29)	Reduction
Northeast II	-0.019 (-0.076-0.037)	0.819	0.463	-4.36 (-15.85-8.70)	Stability
North	-0.055 (-0.074− -0.035)	0.963	< 0.001	-11.97 (-15.71− -8.06)	Reduction
West I	-0.121 (-0.154− -0.098)	0.947	< 0.001	-24.35 (-26.94− -21.67)	Reduction
West II	-0.096 (-0.120− -0.072)	0.963	< 0.001	-19.94 (-24.08− -15.56)	Reduction
Pirineus	-0.058 (-0.102− -0.014)	0.941	0.015	-12.52 (-20.73− -3.46)	Reduction
Rio Vermelho	-0.050 (-0.094− -0.005)	0.950	0.031	-10.93 (-19.39− -1.58)	Reduction
Serra	-0.091 (-0.118− -0.065)	0.948	< 0.001	-19.08 (-23.78− -14.08)	Reduction
Southwest I	-0.045 (-0.057− -0.033)	0.807	< 0.001	-9.87 (-10.87− -8.86)	Reduction
Southwest II	-0.060 (-0.097− -0.023)	0.984	0.005	-13.01 (-19.88− -5.55)	Reduction
South	-0.02 7 (-0.041− -0.012)	0.966	0.002	-6.06 (-9.11− -2.91)	Reduction
São Patrício I	-0.075 (-0.101− -0.048)	0.942	< 0.001	-15.89 (-20.74− -10.73)	Reduction
São Patrício II	-0.031 (-0.067− -0.004)	0.512	0.078	-6.97 (-14.1 1-0.77)	Stability

a Regression coefficient.

b 95% confidence interval.

When analyzing the correlation between HPCSC rates and FHS coverage in Goiás, we verified a negative correlation for the Northeast II (r = 0.464; p = 0.151) and São Patrício II (r = 0.714; p = 0.014) regions, and a positive correlation to the other regions, according to Table 4.

**Tabela 4:** Correlação entre taxa de internações por condições sensíveis à atenção primária (ICSAP) e cobertura da Estratégia Saúde da Família (ESF) em Goiás, por região de saúde, 2005-2015.

Region	HPCSC rate	
	r^a^	p
Goiás	-0.919	< 0.001
Central	-0.839	0.001
Center-South	-0.803	0.003
North Surroundings	-0.857	0.001
South Surroundings	-0.856	0.001
Estrada de Ferro	-0.936	< 0.001
Northeast I	-0.948	< 0.001
Northeast II	-0.464	0.151
North	-0.882	< 0.001
West I	-0.953	< 0.001
West II	-0.872	< 0.001
Pirineus	-0.806	0.003
Rio Vermelho	-0.751	0.008
Serra	-0.931	< 0.001
Southwest I	-0.844	0.001
Southwest II	-0.944	< 0.001
South	-0.732	0.010
São Patrício I	-0.889	< 0.001
São Patrício II	-0.714	0.014

a Coeficiente de correlação de Pearson.

## DISCUSSION

The HPCSC had a significant reduction trend in the state of Goiás over the analyzed period, verified in most health regions. Also considering the hospitalizations due to other conditions, we observed that the greatest decline occurred in the PCSC. Despite this progressive decrease, this rate remained high for the parameters established by the MS, and the reduction trend was not linear for all causes^10^ ^,^ ^20^. Thus, when drawing a general panorama of the PCSC by sex and region of the state, the data from this study allow the development of public policies, in particular regarding PHC.

Recent research conducted in Brazil have shown important reduction of HPCSC, correlating them to factors such as the expansion and strengthening of the PHC, especially from the implementation of the FHS^19^ ^,^ ^21^ ^,^ ^22^.

As it is a relatively new indicator in the scenario of national research, studies evaluating HPCSC trend indicate the absence of a pattern of most prevalent causes, thus presenting a great heterogeneity in the ranking of these injuries^19^ ^,^ ^21^ ^–^ ^23^.

In this study, when evaluating the five most prevalent HPCSC diagnosis groups, we observed that they were the same for both sexes (gastroenteritis, heart failure, lower airway diseases, asthma, and kidney and urinary tract infections), with variation concerning the order of prevalence. A study conducted by Alfradique et al.^6^, which drafted the Brazilian list of PCSC, evaluated the admissions in the year 2006 and presented similar results, except for the group of kidney and urinary tract infections. In Pernambuco, Mendonça and Albuquerque^22^ refer to a broad heterogeneity in the studied health regions and associate the decline in the HPCSC with investments that are being increasingly made in FHS.

In the analysis of this indicator, it is valid to point out essential role of PHC. Some causes of the prevalence of these diagnosis groups are their low resolution, difficult access to the health care system, lack of financial investment in strategic areas, and low resources’ qualification^19^ ^,^ ^22^. In this perspective, research that sought to assess PHC in Brazil showed that access to SUS has been the most critical difficulty to the system, understanding that access inequalities are a barrier to the consolidation of PHC as a gateway^24^ ^,^ ^25^. When the community is unable to access the service, or when it does not find a team able to accommodate their demands, the HPCSC tend to maintain high rates, and some diagnosis groups may be more prevalent, which leads to higher expenses for the health system^19^ ^,^ ^26^.

Despite advances in the implementation of public policies in the area of health, these factors show that a fragmented system (as the Brazilian is), in which levels of attention do not communicate and care is oriented to acute conditions and worsening of chronic conditions, is still not able to respond efficiently to the current situation, which reflects negatively on the studied indicator^2^ ^,^ ^27^ ^,^ ^28^.

When evaluating the trend of HPCSC rates, we observed a reduction in most diagnostic groups - some more pronounced, as in the case of hypertension, gastrointestinal ulcers, and asthma. The literature estimates that lower rates of HPCSC are associated with factors such as good basic care coverage,greater number of general physicians per inhabitants, and improvement of the population's health, as well as effective public policies with a focus on integral care and investments in actions of health promotion and disease prevention^19^ ^,^ ^29^ ^,^ ^30^. The data from the MS to Goiás in 2015 showed that the coverage of basic care was of 69.0% and that there were 1.58 general physicians per 1,000 inhabitants in 2014, which can be considered satisfactory in comparison to other national means (considering the sociodemographic characteristics of the state). The Gini index, which measures social inequality, was 0.52, and the human development index of the state was 0.731^16^ ^,^ ^31^.

Among the groups of causes that did not follow the reduction trend, it is valid to mention the group of immunopreventable diseases, which, despite the stability, presented the second lowest percentage in frequency among the HPCSC. Studies showed that, since the implementation of the *Programa Nacional de Imunização* (PNI - National Immunization Program), various measures have been adopted to achieve a wide immunization coverage, such as the increase in the number of vaccine rooms and improvement of the program's information system, in addition to the implementation of new immunobiological substances in the routine calendar and national campaigns^32^ ^,^ ^33^. As well as the PNI, it is important to highlight that public policies implemented in the last decades have significantly contributed to modify the population's morbidity and mortality profile. Other examples can be cited, such as the *Programa de Assistência Farmacêutica* (Pharmaceutical Assistance Program) and the *Programa Hiperdia* (Arterial Hypertension Program).

However, it is not enough that the programs are implemented, it is necessary to ensure their complete and efficient operation. Mendes^27^ claims that health systems should be structured to face specific conditions. To this end, a complete cycle of care is necessary, starting in primary care and referral to the other levels of care, seeking to ensure integral care, the so-called *Rede de Atenção à Saúde* (RAS - Health Care Network).

When considering the trends in HPCSC rates in the health regions of the state of Goiás, we observed a reduction in almost all, with stability in the Northeast II and São Patrício II regions. Data from Goiás Health Map show that the Northeast II region (one of the most distant from the capital) comprises 11 municipalities and has a total population of 100,179 inhabitants. The São Patrício II region encompasses eight municipalities, totaling 167,391 inhabitants. They have two of the lowest municipal human development indexes (MHDI) and low SUS performance index (IDSUS). The Northeast I region, on its hand, has one of the highest poverty indexes. These sociodemographic factors can negatively influence the studied indicator^16^.

The Brazil HPCSC Project points out in its report that the Brazilian state with the lowest percentage of coverage by Community Health Agents and by FHS was Goiás, with 55.6% and 48.1%, respectively, and that states with better FHS coverage had better reductions in HPCSC rates^21^. Regarding the influence of FHS in reducing HPCSC rates presented in this study, it is necessary to emphasize the positive correlation in all groups of causes, as shown in other studies, associating the reduction of rates with improved FHS coverage^21^.

Limiting factors deserve to be highlighted, such as the restricted scope of the HPCSC index (only SUS hospitalizations), the register or sub-register of the hospitalizations and possible flaws in diagnostic classification.

Temporal trends analyses of HPCSC in Goiás points to the reduction of rates during the investigated period, a result that confirms what has been observed in other states. However, further studies should be performed to enlarge the understanding of other factors (demographic and socioeconomic), seeking to evaluate their relationship with HPCSC.

It is important to monitor this indicator as a starting point to direct actions regarding primary health care, with a view to its potential to measure the quality of health services and to identify critical points that deserve intervention, as well as assessing whether the strategic actions implemented in the community have been effective, considering the modification of the population's morbidity and mortality profile.
